# Banana and Guava dataset for machine learning and deep learning-based quality classification

**DOI:** 10.1016/j.dib.2024.111025

**Published:** 2024-10-18

**Authors:** Abiban Kumari, Jaswinder Singh

**Affiliations:** Department of Computer Science and Engineering, Guru Jambheshwar University of Science and Technology, Hisar 125001, Haryana, India

**Keywords:** Fruit classification, Computer vision, Image processing, Machine learning, Deep learning

## Abstract

In the field of agriculture, the identification and classification of fruits have become necessary for sustainable growth in horticulture sectors. Recently, different types of advanced techniques have been used for the identification and classification of fruits. Machine learning is an emerging technology with robust application in different sectors that can be used for the classification of fruits. The basic requirement for the machine learning techniques is the availability of the dataset to develop a robust machine learning model. But the limited availability of the data set is one of the major challenges in this sector. The classification of fruits using non-destructive methods can be executed through the availability of a comprehensive dataset. The rapid and precise classification of the fruit according to their quality and maturity is the desired need of the fruit storage, processing, and export industries. Therefore, this article provides a comprehensive dataset for fruits (banana and guava) and their classification according to their quality classes. The dataset was acquired using a Redmi Note 10-Pro mobile camera in natural sunlight at different angles for different classes in JPG format. The acquired dataset was classified into three different categories, Class A, Class B, and Defect, based on their physiological changes. The obtained datasets can be used for the development of different machine learning models for the quality classification of banana and guava.

Specifications Table

**Table:** 

Subject	Computer science, Agriculture science.
Specific subject area	Computer vision, Maturity analysis, Quality classification
Type of data	Fruits Images
Data collection	The images of fruits were captured using mobile phones camera with high resolution. The original .jpg images were obtained with dimensions of 4640 × 3472, which were further resized to 1024 × 1024 dimensions.
Data source location	Location: Guru Jambheshwar University of Science and Technology Zone: Hisar, Haryana Country: India
Data accessibility	Repository name: Mendeley data [1] Data identification number: 10.17632/56td5w4wz2.2 Direct URL to data: https://data.mendeley.com/datasets/56td5w4wz2/2
Related research article	None

## 1 Value of the Data

• *Automated Browning Monitoring System:* The dataset provides a foundation for the future research in the area of physiological mechanism and behaviour of the fruits underlying the enzymatic browning. The quality of the fruits affects the consumer acceptability, fruits storage shelf life and the quality of the developed products. The nutritional quality of the climacteric fruits (banana and guava) is continuously changes as the fruits enter from the ripe stage to the rotten stage. In the later stage of ripening, the fruits show brown patches on the peel or outer surface due to the degradation of the yellow color pigment, which ultimately decrease the postharvest shelf life and reduce the fruit quality. Thus, the data can be used for the development of automated system to determine the initiation of fruit browning.
• *Fruit Quality During Accelerated Storage:* The dataset can be used to determine the fruits quality at ambient, controlled and accelerated storage, which will be beneficial for, farmers, storage industries (for long term storage), processing industries (to deliver good quality products with better shelf life) and export industries. Because, the ripening is continuous process in the climacteric fruits and peel color changes continuously with the storage time due to different physiological and biochemical changes. Thus, the dataset will help to overcome the challenges occur during the fruit classification.
• *Development of Precise Computer Vision Model*: The data can be used for the training and testing of different models to increase their accuracy and precision. The determination of fruits freshness, maturity level and defect in banana and guava is essential for the fruit's quality classification, to reduce the postharvest losses and develop an avenue for the national and international market. The acquired comprehensive datasets plays an important role in this endeavor by providing an asset for the computer vision model.
• *Development of Automated System:* The datasets have the huge potential for the development of an automated service-oriented system for postharvest classification of fruits (Banana and Guava) using computer vision techniques. The developed system will provide a consistent and non-biasness fruit classification. So, that the farmers and fruit processing, storage and export industries can strategically identify the exact postharvest storage time of the fruits, and sale their product accordingly at good price. It will help to reduce the postharvest losses at the farm and industrial level. Thus, the automated system will help to deliver an accurate classification by image analysis and help to overcome the major challenges faced during fruit classification at the farm and industrial levels.

## 2 Background

Fruits are an important horticulture commodity, generally known for its delicious taste and nutritional value. India is the largest producer of the banana and guava. The high perishability, high postharvest losses and subjective classification are the major hurdle in the quality classification of the fruits. Therefore, an automated system is required for the classification of the Banana and Guava. But the availability of fruit dataset with consistent images is the major limitation in the horticulture crops, which hinder the development of precise and rapid automatic system for the fruit classification. The proposed dataset provides a distinct advantage with different angles and color over the already available dataset in different data repository. In the present study, the dataset was specifically designed to address the challenges in the development of an automatic system. The dataset presented in the current study is completely different or novel from the already available dataset. The acquired datasets are focused on the postharvest quality (browning stages) of fruits (Banana and Guava), which have not been included in any of the data available in the literatures. The main motivation behind the collection of this dataset specifically for (Banana and Guava) was the high postharvest losses in both of these crops. Recent studies showed more than 25% losses due to postharvest physiological and biochemical changes such as enzymatic degradation.

The datasets (banana and guava) available in the literature including DiB is focused on the classification of the fruit in green, mature green and ripe stage [1], Immature, half matured and matured [2]; fresh and rotten images [3]; good, bad and mixed quality [4], disease (red rust, scab and styler end rot) classification [5]; tier-based classification [6], fruit variety identification [7,8]. To the best of our knowledge, we have not found the dataset focusing on the postharvest physiological changes such as enzymatic browning, as presented in the current study. Therefore, the dataset presented in the study is novel and different from already available studies. The main rational behind the collection of data together for Banana and Guava, is the climacteric nature of these fruits. The both banana and guava are come in the category of climacteric fruits and follow the similar pattern climacteric rise such as increase in the respiration rate, increase endogenous ethylene, flavor development, cell wall degradation and color changes from yellow to light brown and further dark brown in the later stages. Therefore, the main objective of the study is to provide a comprehensive dataset for the development of an advanced machine learning model for non-destructive classification of fruits.

## 3 Data Description

**Fig. 1:**
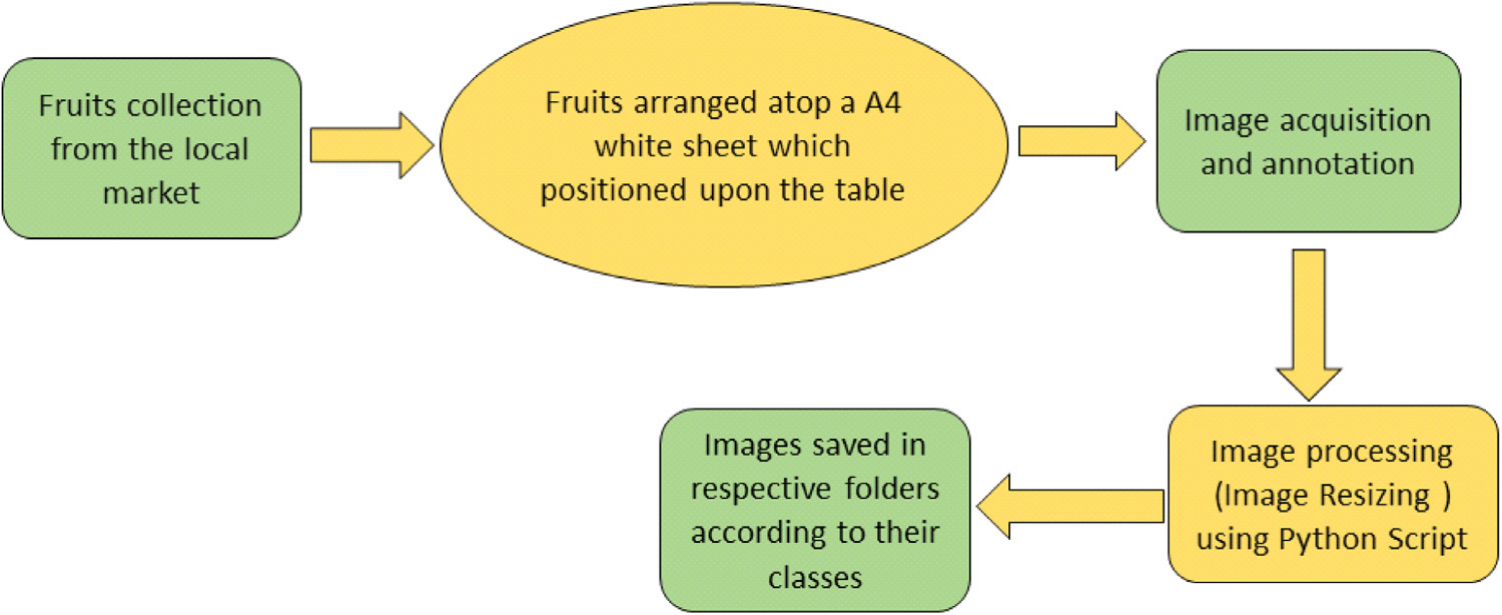
Detailed process for the image acquisition.

The acquired banana and guava images dataset can serve for different purpose, including the identifications of fruit maturity level and their quality classification for the storage and export purpose. The dataset contains total 1738 images of banana and guava in three different classes. The images dataset was classified as; a) Class A, b) Class B, and c) defect quality, according to the maturity stage of the fruits. The dataset was acquired from the different angles and sides to obtained the high accuracy during model development. The dataset was further stored in different folders and further subdivided into subfolders according to their classes. Moreover, each folder contains three subfolders with Class A, Class B, and defect quality for banana and guava. The detailed method of image acquisition process is presented in Fig. 1.

## 4 Experimental Design, Materials and Methods

The processing of the dataset was performed in two different steps as discussed below:

1. *Image Acquisition and Annotation*: The fruits were procured from the local fruit market, Hisar, Haryana, India. The images were acquired using Xiaomi (Redmi Note 10-Pro) mobile with 108 MP(Mega-Pixel) camera. The fruit images were captured in natural sunlight. All images were captured wsith different angles and saved in JPG format. The detailed specification of the camera is presented in Table 1.

**Table 1:** Detailed specification of data acquisition tool.

Sr. No.	Camera parameter	Description
1	Maker	Xiaomi (Redmi)
2	Model	M2101K6P
3	F-stop	f/1.8
4	Time of exposure	1/50 s.
5	ISO speed	ISO-56
6	Exposure bias	0 steps
7	Length of focal	5 mm
8	Max aperture	1.67
9	Metering mode	Center weighted average
10	Flash	No flash

The annotation of data was performed by two ways;

(a) The annotation was performed in the presence of expert in the field of postharvest physiology of the fruit specially in climacteric fruits.
(b) The annotation was further confirmed by the Technical Guide from Food and Agriculture Organization (FAO), United State and the previously published research on fruit ripening behavior [[9], [10], [11], [12]]. The acquired dataset was stored in their respective folder and further used for preprocessing.

2. *Image Processing:* Initially, the acquired images of the banana and guava were separated. The acquired images were categorized into three classes; a) Class A, b) Class B, and c) Defect Quality (Table 2). The simages were acquired with the size of 4640 × 3472 pixels. The total number of acquired original images were 1738 and the detailed specification is provided in Tables 3 and 4.

**Table 2:** Details of different classes of banana and guava.

Fruit type	Class name	Description	Image
Banana	Class A	Class A of banana can be recognized by its uniform color, appearance, texture and good firmness. The best quality of banana (Class A) typically identified by a bright yellow color.	
	Class B	Class B of banana can be recognized by yellow color with minor blemishes, slight brown spot and slightly soft texture and firmness as compared to the class A of banana.	
	Defect	The defect class of banana can be recognized as significant discoloration (multiple large brown spot) and unacceptable fruit firmness.	
Guava	Class A	Class A of guava can be identified by its uniform color, appearance, texture and firmness. The best quality of guava (Class A) typically identified by a bright yellow color.	
	Class B	Class B of guava can be recognized by yellow color with minor blemishes, slight brown spot and slightly soft texture as compared to the class A of guava.	
	Defect	The defect class of guava can be recognized as significant discoloration (multiple large brown spot), unacceptable soft texture and rotten spot.

**Table 3:** Detailed description about the fruit images.

Sr. No.	Particulars	Details as per Fruit Classes		
		Class A	Class B	Defect
1	Dimension	1024 × 1024	1024 × 1024	1024 × 1024
2	Width	1024 × 1024	1024 × 1024	1024 × 1024
3	Height	1024 × 1024	1024 × 1024	1024 × 1024
4	Horizontal Resolution	96 dpi	96 dpi	96 dpi
5	Vertical Resolution	96 dpi	96 dpi	96 dpi
6	Bit Depth	24	24	24
7	Resolution unit	2	2	2
8	Color representation	sRGB	sRGB	sRGB

sRGB- Standard Red Green and Blue.

**Table 4:** The number of images in different classes of the banana and guava.

Sr. No.	Fruit Type	Number of images as per Fruit Classes			Total
		Class A	Class B	Defect	
1	Original Banana	509	281	403	1193
2	Original Guava	161	179	205	545
3	Total	1738			

Further, the image dataset was processed [2] using python script to resize the image pixels into 1024 × 1024. The detailed description about the different classes is provided in Table 3.

## Limitations

The limitation of the dataset is that the image dataset was generated for two types of fruits (Banana and Guava), which may hamper the design of robust machine-learning models for different kinds of fruits. Because the dataset is limited to only banana and guava. Therefore, the study encourages the researchers to get more datasets for various types of climacteric and non-climacteric fruits for non-destructive classification.

## Ethics Statement

For the preparation of the dataset, there was no human or animal experimentation involved.

## Credit Author Statement

**Abiban Kumari:** Software, Validation, Methodology, Writing – original draft; **Jaswinder Singh:** Supervision, Writing – editing and review.

## Data Availability

Mendeley DataFruits (Banana and Guava) datasets for non-destructive quality classifications (Original data).
